# Data demonstrating distinct embryonic developmental defects induced by bisphenol a alternatives

**DOI:** 10.1016/j.dib.2019.104091

**Published:** 2019-06-03

**Authors:** Ashley L. Arancio, Emry R. Cohenour, Kyla D. Cole, Anyssa R. Dominguez, Julia Kadie, William Cooper Maloney, Chane Cilliers, Sonya M. Schuh

**Affiliations:** Department of Biology, School of Science, Saint Mary's College of California, 1928 Saint Mary's Road, Moraga, CA 94576, USA

**Keywords:** BPA, BPAF, BPA alternatives, 17-β estradiol, Embryo, Development

## Abstract

Embryos of *Xenopus laevis* (African clawed frog) were exposed to the widespread environmental plasticizers bisphenol AF (BPAF; 0.003–3 μM), bisphenol A (BPA; 1–50 μM), or 17β-estradiol (E2; 10 μM) from just after fertilization through 96 hours of development. The potencies and cellular and morphological effects were compared across chemical treatments and controls. The embryos were staged, counted and imaged, and time-lapse movies collected, on an inverted stereomicroscope and camera. The data show there were both shared and unique effects of BPAF, BPA, and E2, on early cleavage divisions and development of the spinal cord, head, and gut, with BPAF having the greatest potency and toxicity (1000 times more potent than BPA). Specifically, cleavage divisions, within 1–6 hours of exposure had severe irregularities including asymmetrical division, slowed mitosis and cytokinesis, cellular dissociation, and fewer numbers of cells per embryo. By 48 hours of exposure the embryos had curved body axis defects, neural tube defects including curved, incomplete, or two neural tubes, ventral and gut blisters, and overall extreme abnormalities. By 96 hours of exposure estradiol caused tail flexures/bent spines, severe pigmentation reduction, long loosely coiled gut, and a ventral blister in 100% of embryos. BPA caused truncated body axis defects, tail flexures, and head and eye malformations in over 60% of embryos. BPAF, at the lowest doses tested, caused craniofacial defects, shorter tails, ventral blisters, edema and peritoneal effusion in over 75% of the surviving embryos. For a complete description, interpretation of the data and a discussion refer to the article in press Arancio et al., 2018.

**Table:** Specifications table

Subject area	Biology
More specific subject area	Developmental and reproductive toxicology
Type of data	Images (microscopy), figures, videos
How data was acquired	Leica S6D Stereomicroscope and mounted MC 190 camera (Leica Microsystems, JH Technologies), with a 64-bit Dell Latitude Desktop computer using LAS imaging software (Leica Microsystems)
Data format	Raw data
Experimental factors	Exposure to BPAF (0.003–3 μM), BPA (1–50 μM), estradiol (10 μM), or control media (Ringers with 0.03% ethanol)
Experimental features	Embryos were exposed to BPAF, BPA, or estradiol from 1 h post-fertilization to 96 hours of development. The potencies and effects were compared across chemical treatments and controls.
Data source location	Moraga, California, United States
Data accessibility	Data is with this article.
Related research article	Arancio AL,* Cole, KD,* Dominquez AR,* Cohenour ER, * Kadie J,* Maloney WC,* Cilliers C,* and Schuh SM. Bisphenol A, Bisphenol AF, Di-n-butyl phthalate, and 17β-estradiol have shared and unique dose-dependent effects on early embryo cleavage divisions and development in Xenopus laevis, Reprod. Tox., 84, 2018, 65–74 [1].

**Table:** 

**Value of the data** • This data is useful as these BPA compounds are mass-produced and prevalent in our environment, yet very few studies have compared their effects on early embryo development • No studies have examined the effects of BPA and alternatives on cleavage divisions in the earliest stages of development, which corresponds to the window of human development before embryo implantation • Those working in the toxicology, developmental biology and fertility fields can benefit from this data • The general scientific community and public would greatly benefit from learning about these results, as there are direct consequences and implications for human health and disease and the development of better regulations and alternative practices to reduce human exposure • Although used as a BPA alternative in many plastic and consumer products, this data on the potency and toxicity of BPAF, provides greater insight and cause for concern about various plasticizers and manmade chemicals • These data highlight the need for better toxicological characterization of all BPA analogs and encourage collaborative studies comparing the effects across species and at different developmental windows of exposure

## 1 Data

**Fig. 1:**
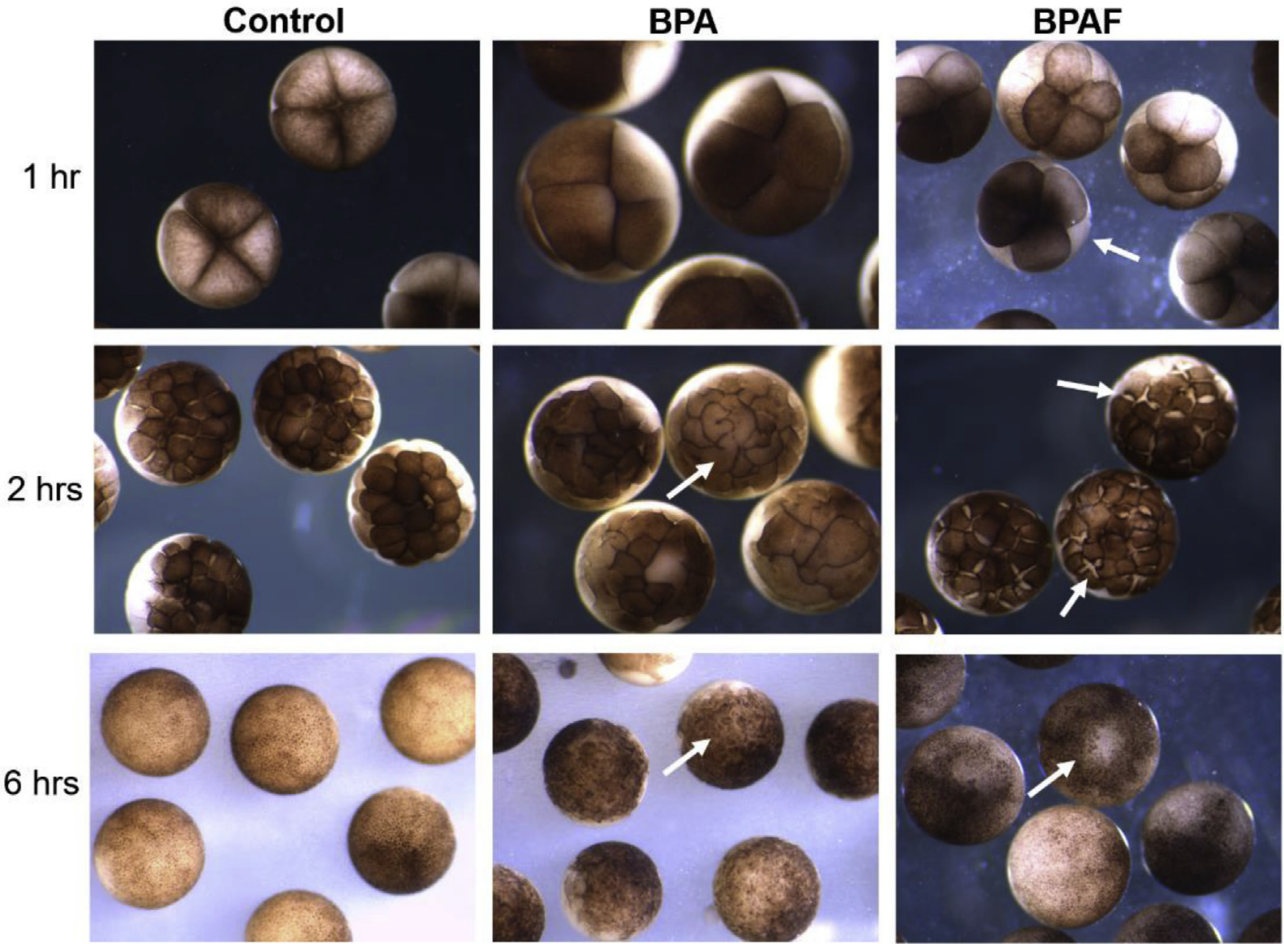
**Early cleavage division defects caused by BPA and BPAF**. Control embryos (left column), compared to embryos exposed to BPA (50 μM; middle column) or BPAF (3 μM; right column) at 1, 2 and 6 hours of exposure. Irregular, asymmetrical mitotic division, slowed cytokinesis, and cellular dissociation were observed in the majority of BPA- and BPAF-treated embryos (arrows).

**Fig. 2:**
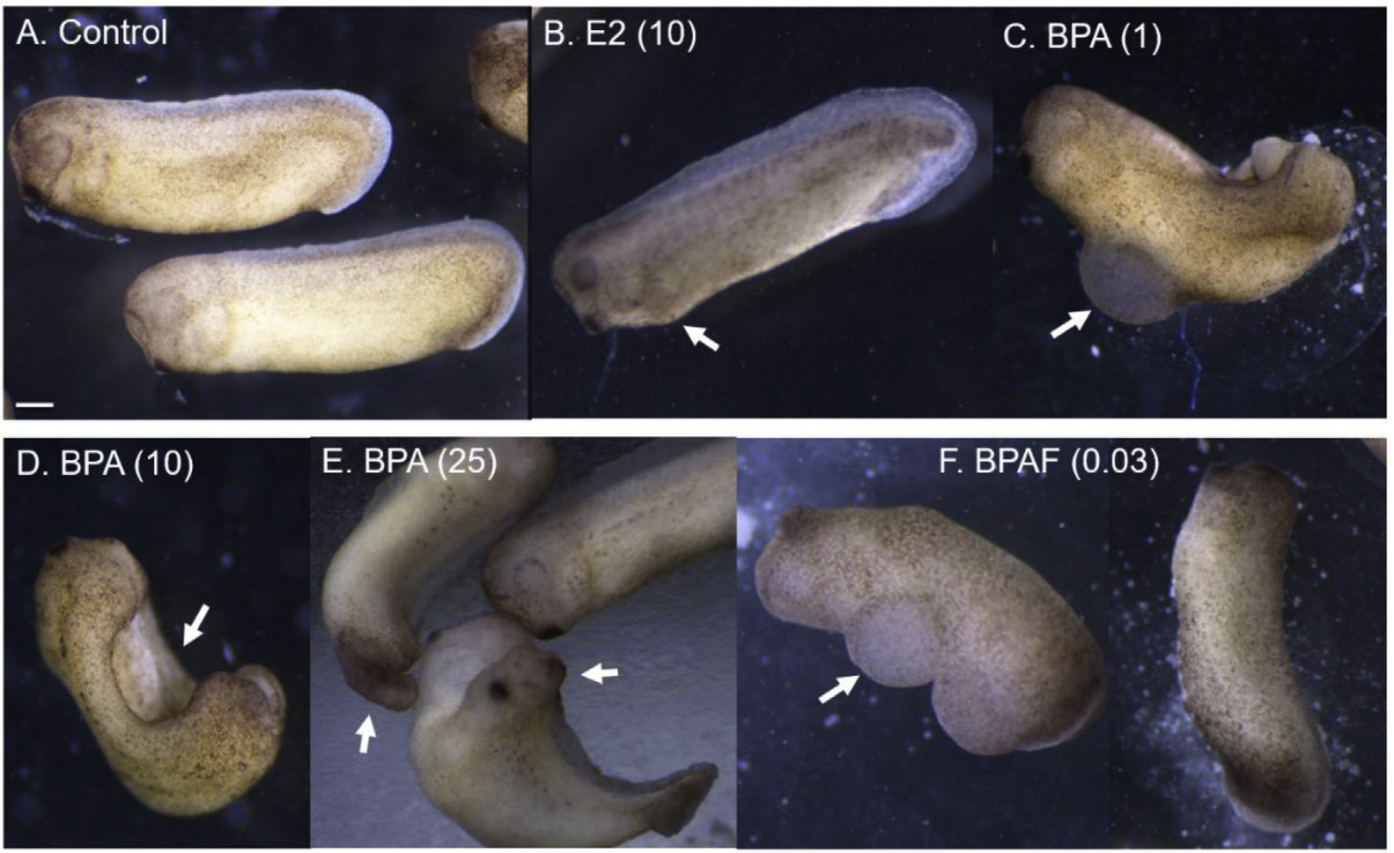
**Early embryological defects caused by E2, BPA, and BPAF**. A) Control tailbud stage embryos at 48 hours of development compared to tadpoles exposed to 10 μM estradiol (B), BPA at 1 (C), 10 (D), and 25 μM (E), and BPAF at 0.03 μM (F), showing curved body axis defects, neural tube defects including two neural tubes, gut blisters, and overall extreme abnormalities (arrows; scale bar = 1 mm).

**Fig. 3:**
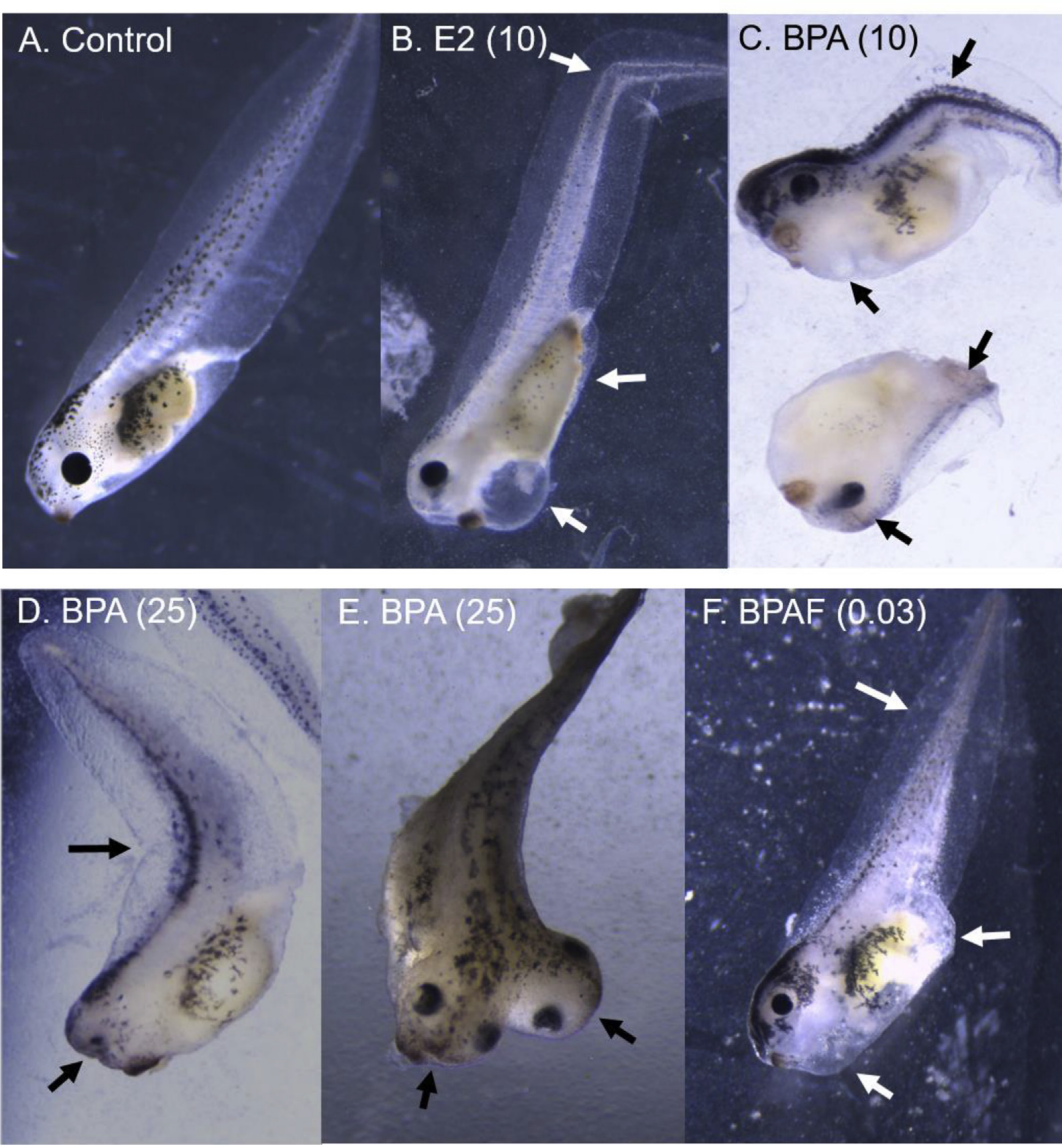
**Late embryological defects caused by E2, BPA, and BPAF**. A) Control tadpole at 96 hours of development compared to tadpoles exposed to 10 μM estradiol (B), BPA at 10 (C) and 25 μM (D, E), and BPAF at 0.03 μM (F). E2 caused tail flexures, severe pigmentation reduction, long loosely coiled gut, and a ventral blister in 100% of embryos (arrows). BPA caused truncated body axis and body axis defects, bent tails, and head and eye defects (C–E, arrows). BPAF caused shorter tails, craniofacial defects, ventral blisters, edema and peritoneal effusion in over 90% of surviving embryos (F, arrows).

We exposed *Xenopus* embryos to various doses of BPAF, BPA, or estradiol from 1–96 hours of development and compared them to control embryos bathed in Ringers media (with 0.03% ethanol). The embryos were staged, counted, imaged, and time-lapse movies collected, on an inverted stereomicroscope and camera. The cellular and morphological effects and potencies were compared across chemical treatments and controls (control embryo at 96 hours showing heartbeat in Video 1). BPA and BPAF severely disrupted early embryonic cleavage within the first 1–6 hours of exposure, causing asymmetrical, slowed mitosis and cytokinesis, cellular dissociation, and reduced cell numbers per embryo (Fig. 1 and Video 2). These effects were not seen with estradiol. Longer BPA and BPAF exposures of 48 hours, resulted in curved body axis defects, neural tube defects including incomplete and two neural tubes, gut blisters, and overall extreme abnormalities (Fig. 2). Surviving tadpoles exposed for 96 hours to 10 and 25 μM BPA, 30 nM BPAF, or 10 μM estradiol had various morphological defects. E2 caused tail flexures and bent spines, severe pigmentation reduction, long loosely coiled gut, and a ventral blister surrounding the pericardial sac in 100% of embryos (Fig. 3B, arrows; Video 3). BPA caused truncated body axis and body axis defects, tail flexures, and head and eye defects (Fig. 3C–E, arrows). BPAF caused shorter tails, craniofacial defects, ventral blisters, edema and peritoneal effusion in over 90% of surviving embryos (Fig. 3F, arrows).

## 2 Experimental design, materials and methods

### 2.1 Collection and preparation of embryos

Adult *Xenopus laevis* were maintained on a diurnal cycle (14 hr light:10 hr dark) at the University of California, Berkeley, Life Sciences Animal Facility as described previously [1]. All experiments were approved and performed in strict accordance with the Institutional Animal Care and Use Committee of U.C. Berkeley and Saint Mary's College and were conducted following the Guidelines for the Use of Live Amphibians and Reptiles in Field and Laboratory Research [2]. Adult males and females, bred in the laboratory, were given a single injection of 450 IU and 750 IU human chorionic gonadotropin, respectively, in the dorsal lymph sac. Eggs were obtained 24 hours later manually by the squeeze technique. Testes were dissected from adult male frogs and stored for up to two weeks at 4 °C for in vitro fertilization (IVF) use. To perform IVFs, a small fragment (1–2 mm) of testis was minced to create a sperm suspension. The eggs and sperm were flooded with 0.1X MBS (Modified Barth's Saline) and fertilization time was recorded. Thirty minutes after fertilization, the embryos were dejellied with a 3% cysteine solution (Sigma-Aldrich, St Louis, MO) for 2–3 minutes and then rinsed several times with 0.1X MBS. Unfertilized and necrotic eggs were removed, and healthy fertilized embryos at the two-cell stage were selected 2 hours post-fertilization (h.p.f.) according to the *Xenopus laevis* Nieuwkoop stages of development [3].

### 2.2 Media and chemical exposures

Stock solutions of BPA (Cat. No. 133027;-500G; >97% purity), BPAF (Cat. No. 90477; >99% purity), and E2 (Cat. No. E8875-1G; > 98% purity) were prepared in 95% ethanol or DMSO (Cat. No. 0231–500 ml; purity >99.9%) in glass bottles. Final test solutions were made by diluting the stocks in 0.1X MMR solution (Modified Marc's Ringer). Ethanol/DMSO (0.03%) was also added to the 0.1X MMR control solutions (all final control and test solutions contained 0.03% ethanol/DMSO). The 0.1X MMR working stock was diluted from a 10X stock of 0.1 M NaCl, 2 mM KCl, 1 mM MgSO_4_, 2 mM CaCl_2_, 5 mM HEPES (pH 7.8). For IVFs, a 0.1X MBS, was diluted from a 10X stock (88 mM CaCl_2_, 1 mM KCl, 1 mM MgSO4, 5 mM HEPES, 2.5 mM NaHCO^3^, pH 7.8) and a High Salt MBS medium was used (7 ml 0.1 M CaCl_2_, 100 ml 10X MBS salts, 4 ml 5 M NaCl, in 888 ml water, pH 8). All chemicals were purchased from Sigma-Aldrich (Sigma-Aldrich, St. Louis, MO).

The embryos were divided up evenly into glass dishes (50 mm, Corning, New York) containing control or treatment solutions approximately 2 h.p.f.. Static renewal of all solutions was performed every 12–24 hours and dying or necrotic embryos were aspirated out. The experiments were performed beginning at the two-cell embryo stage with the concentrations of: BPA: 0, 1, 10, 25, and 50 μM (scale of ppm); BPAF: 0, 0.003, 0.03, 0.3, 3, and 25 μM (ppb–ppm); and E2: 10 μM (ppm). Approximately 25–50 eggs or embryos were bathed in each of the control or treatment solutions, and run in duplicate or triplicate (a total of ∼100 embryos per treatment per trial). All trials were independently replicated three to ten times, with a total of ∼300–1000 embryos per treatment, using eggs from 6–20 adult female frogs.

### 2.3 Microscopic imaging and developmental staging

Surviving embryos were observed, counted, staged, and imaged at 22 °C for up to 96 hours after fertilization to define the morphological abnormalities. Staging and patterning of abnormal development were performed and recorded as described by Nieuwkoop and Faber [3]. Dead embryos were not counted as malformed/abnormal. There are variable rates of embryo mortality that occur even in control embryos and vary between batches. Therefore, when the embryonic survival rate of the control groups at 96 h.p.f. was lower than 85%, then the developmental toxicity data were discarded. Heartbeat was used to confirm survival (Video 1). Embryos were carefully analyzed and imaged at 0, 1, 2, 4, 6, 48, and 96 hours. Images were captured using an inverted Leica Stereomicroscope and mounted camera (Leica Microsystems, JH Technologies, Fremont, CA), with a 64-bit Dell Latitude Desktop computer using LAS imaging software (Leica Microsystems). All counts and staging were performed independently by two different observers, and averaged when differences in counts were recorded.
